# Assessment of the adverse pregnancy outcomes and its associated factors among deliveries at Debre Berhan Comprehensive Specialized Hospital, Northeast Ethiopia

**DOI:** 10.1371/journal.pone.0271287

**Published:** 2022-07-08

**Authors:** Mesfin Tadese, Kefyalew Dagne, Abate Dargie Wubetu, Shiferaw Abeway, Alemayehu Bekele, Worku Misganaw Kebede, Getaneh Baye Mulu

**Affiliations:** 1 Department of Midwifery, College of Health Sciences, Debre Berhan University, Debre Berhan, Ethiopia; 2 Department of Psychiatry, College of Health Sciences, Debre Berhan University, Ethiopia; 3 Department of Psychiatry, School of Medicine, College of Health Sciences, Addis Ababa University, Ethiopia; 4 School of Nursing and Midwifery, Department of Pediatrics and Child Health Nursing, College of Medicine and Health Sciences, Wollo University, Dessie, Ethiopia; 5 Ethiopian Public Health Association, Research, and Publication Directorate, Addis Ababa, Ethiopia; 6 Department of Nursing, College of Health Sciences, Debre Berhan University, Debre Berhan, Ethiopia; National Research Centre of Egypt, EGYPT

## Abstract

**Background:**

Adverse pregnancy outcomes are the main causes of maternal and neonatal morbidity and mortality and long-term physical and psychological sequels in low- and middle-income countries, particularly in Africa and Asia. In Ethiopia, maternal mortality remained high despite the country’s maximum effort. This study aimed to assess adverse pregnancy outcomes and associated factors among deliveries at Debre Berhan Comprehensive Specialized Hospital, Northeast Ethiopia.

**Methods:**

A retrospective cross-sectional study was done among deliveries at Debre Berhan Comprehensive Specialized Hospital from January 1, 2017, to December 31, 2018. The data was collected using a structured and pre-tested questionnaire by reviewing labor and delivery service log books and admission or discharge registration books. The data were entered into a Microsoft Excel spreadsheet and analyzed using SPSS version 25. Logistic regression analysis was computed to identify independent predictors of pregnancy complications.

**Result:**

In this study, the magnitude of adverse pregnancy outcomes was 28.3%, 95% CI (25.7–30.9). The most frequently recorded obstetric complications were obstructed labor (7.4%), retained placenta (5.3%), and hypertensive disorders of pregnancy (2.4%). Whereas stillbirths (10%), malpresentation (3%), and prematurity (2.3%) frequently occurred fetal/neonatal complications. There were 29 maternal deaths and the possible causes of death were obstructed labor (51.7%), hemorrhage (44.7%), eclampsia (24.1%), and sepsis (6.9%). Home delivery (AOR (CI = 4.12 (2.30–7.15) and low birth weight (AOR (CI = 1.63 (1.36–1.96) were significant associates of adverse pregnancy outcomes.

**Conclusion:**

The magnitude of adverse pregnancy outcomes was high. Obstructed labor, retained placenta, hypertension in pregnancy, malpresentation, prematurity, and stillbirth are the commonest adverse pregnancy outcomes. Place of delivery and birth weight were independent predictors of adverse pregnancy outcomes. Institutional delivery, early detection and management of complications, and adequate nutrition and weight gain during pregnancy should be encouraged to minimize the risk of adverse pregnancy outcomes.

## Introduction

Pregnancy outcome also called birth outcome, is the final result of fertilization events that occur to the newborn infant from the age of viability (28 weeks) to the first weeks of life. These outcomes vary from pregnancy to pregnancy and include live birth (full-term or preterm birth), stillbirth/intrauterine fetal death, spontaneous abortion, induced abortion, and early neonatal death [1]. Adverse pregnancy outcome is a broad term comprising health problems that occur to the mother, the newborn, or both during pregnancy, labor and delivery, and the postpartum period. Some of the common pregnancy complications include antepartum hemorrhage (APH), hyperemesis gravidarum, postpartum hemorrhage (PPH), stillbirth, low birth weight, premature rupture of membranes (PROM), obstructed labor, hypertensive disorders of pregnancy, prematurity, uterine rupture, and puerperal sepsis [2]. Thus, women must receive health care before and during pregnancy to decrease the risk of adverse pregnancy outcomes.

According to the World Health Organization (WHO) reports, every day approximately 810 women die of preventable complications during pregnancy, childbirth, or postpartum period globally [3]. In 2017, more than 295,000 women died during and following pregnancy and childbirth. The vast majority (94%) of all deaths occur in low and lower-middle-income countries such as Ethiopia. Sub-Saharan Africa accounted for nearly 66% (196,000) of the global maternal deaths, while Southern Asia accounted for about 20% (58,000) [3]. However maternal mortalities only tell part of the story. For every woman who dies of pregnancy-related causes, between 20 and 30 more will suffer short- and long-term disabilities, such as obstetric fistula, infections, a ruptured uterus, or pelvic inflammatory disease [4].

Despite all measures and efforts, Ethiopia’s maternal mortality rate (MMR) continues to be high and alarming. According to the Demographic Health Survey 2016 (DHS-2016), the maternal mortality rate (MMR) in Ethiopia is 412 per 100,000 live births and the neonatal mortality was 29 per 1000 live births [5]. Over 25,000 women and girls die each year due to pregnancy-related complications. Besides, more than 500,000 Ethiopian women and girls will suffer from disabilities caused by complications during pregnancy and childbirth each year [4]. The main direct causes of maternal death in Ethiopia include pregnancy complications, i.e., hemorrhage (29.9%), obstructed labor/ruptured uterus (22.3%), pregnancy-induced hypertension (16.9%), puerperal sepsis (14.68%), and unsafe abortion (8.6%) [6].

The burden of adverse pregnancy outcomes in low- and middle-income countries is still high. In Nepal, 27.8% of women had adverse obstetric symptoms during the intrapartum period [7]. The prevalence of adverse birth outcomes in Sub-Saharan Africa is 29.7% [8]. One-third of reproductive-age women in Uganda reported at least one adverse pregnancy outcome [9]. Similarly, in Zimbabwe, 15.61% of women experienced an adverse pregnancy outcome [10]. The magnitude of adverse birth outcomes was 24.5% in Hosanna town [11], 21% in Bale Zone [12], 18.3% in Hawassa town [13], and 32.5% in Dessie Referral Hospital [14]. In North Gondar, 28.5% of women reported some kind of obstetric complications. Excessive bleeding and prolonged labor were the commonest complications [15]. Similarly, in Southern Ethiopia, hypertensive disorders of pregnancy, APH, PROM, and obstructed labor were the commonest adverse obstetric outcomes [16]. The most frequently identified adverse birth outcomes among newborns were low birth weight, prematurity, and stillbirth [11, 12, 14].

According to a 10-year retrospective review of 7249 deliveries at Adigrat Zonal Hospital, the common obstetric complications identified were preterm labor (7.2%), PROM (6%), pre-eclampsia (5.2%), malpresentation (8.7%), APH (2.7%), and PPH (6.7%). The study further found 32 maternal deaths and the presumed causes were: ruptured uterus (25%), obstructed labor (78.8%), hemorrhage (28.1%), eclampsia (15.6%), and sepsis (12.5%) [17].

Advanced maternal age, low educational status, and early sexual debut were significant associates of adverse pregnancy outcomes [18]. In South Gondar, history of the adverse birth outcome did not receive dietary counseling during pregnancy, and inter-pregnancy intervals less than 24 months were significantly associated with adverse pregnancy outcomes [19]. Age, occupation, residence, and lack of antenatal care were also predictors of adverse pregnancy outcomes in Southern Ethiopia [11]. Further reports in Ethiopia revealed that only 26% of women delivered at health institutions. The majority of births (42%) are attended by a traditional birth attendant at home [5].

Adverse pregnancy outcomes are the most important vital statistics used to assess maternal and child health programs. They are an indicator of the quality of maternal and child health care services, i.e., antenatal care, intrapartum care, and medical services. Since 2000, Ethiopia has reduced maternal and neonatal mortality by half, but a maternal mortality rate of 412 per 100,000 live births and neonatal mortality rate of 29 per 1,000 are still too high [5]. However, most of the causes of these deaths are preventable. Hence, the study aimed to assess the adverse pregnancy outcomes and associated factors among deliveries at Debre Berhan Comprehensive Specialized Hospital, Northeast Ethiopia.

## Methods and materials

### Study area, period, and design

A retrospective cross-sectional study was done from January 1, 2017, to December 31, 2018, at Debre Berhan Comprehensive Specialized Hospital. The hospital is found in Debre Berhan town, which is the administrative center and capital city of the North Shewa Zone of the Amhara region. It is located about 130 km northeast of Addis Ababa on the Ethiopian highway. According to the administration office report, the total population size of the town is 160,408 [20]. Of these, 26.3% of them were reproductive age group women. In the town, 1 Comprehensive Specialized Hospital, 3 health centers, 2 private hospitals, and 17 private clinics were found.

### Sample size, sampling procedure, and study variables

The sample size was calculated using the single population proportion formula with the assumptions of;

◾ P is the population proportion of obstetric complications (P = 32.5%) [14].
◾ Z_⍺/2_ = Z value at (α = 0.05) = 1.96 corresponding to 95% confidence level.
◾ d = the margin of error = 0.02 (conservative estimate)
$$n=\frac{Z_{\frac{\alpha }{2}}^{2}\;p(1-P)}{d^{2}}$$

The resulting sample size was 2103. For possible missing data during the record review, the sample size was increased by 15% and equals 2418. Thus, to fulfill the determined sample size, a two-year retrospective chart review was done.

In the beginning, all maternal birth records and antenatal care files were reviewed. From 4467 identified records, 832 charts were incomplete and 313 charts were missing at the time of data collection and were excluded from the study. Finally, all study participants who fulfilled the eligibility criteria from January 1, 2017, to December 31, 2018, were included.

The primary outcome variable was adverse pregnancy outcome. Other variables of interest extracted from the record review included: age, gravidity, parity, birth weight, place of delivery, number of fetuses, and HIV status.

### Data collection tool and quality control measures

A structured and pre-tested data extraction tool was used to extract the maternal birth records and antenatal care files. The tool was adapted from the Ethiopian Demographic Health Survey questionnaire and other peer-reviewed published literature [5, 18, 19, 21]. Five BSc midwives working in the obstetric department, 2 supervisors, and 1 Ph.D. student were involved in the data collection. The collected data was kept in a secure environment to avoid loss and breach of confidentiality. The principal investigators and supervisors daily cleaned, checked, and stored the collected data.

Data collectors and supervisors were oriented about the objective, method, and data collection procedure and continuously coached by the principal investigator until the end of the data collection period. The advisor (expert in the field of research methods) was also closely consulted. The tool was pre-tested on 5% of records two weeks before the actual data collection time. Following the result of the pre-test, some variables (marital status, ethnicity, occupation, income, body mass index) were deleted from the data collection tool.

### Measurement

#### Adverse pregnancy outcome

The presence of at least one maternal or fetal/neonatal complication i.e., APH, hyperemesis gravidarum, PROM, PPH, preeclampsia, eclampsia, obstructed labor, oligohydramnios, polyhydramnios, low birth weight, stillbirth, macrosomia, malpresentation, meconium aspiration syndrome, prematurity, congenital malformation, puerperal sepsis, preterm delivery, and maternal death [18, 19].

Preterm birth is if the baby is born before 37 completed weeks of gestation but after 28 weeks of gestation. Low birth weight, is when the newborn weight is less than 2500 gm within the first hour of birth, and stillbirth if the infant died in the uterus at or during the intrapartum period at or after 28 weeks of gestation [18, 19]. Parity is the number of births after 28 weeks of gestation. Gravidity is the number of pregnancies regardless of gestational age and outcome.

### Data processing and analysis

Data were cleaned, coded, and entered into a Microsoft Excel spreadsheet and analyzed using SPSS version 25. The descriptive statistics were summarized and presented using tables, graphs, and text. Independent variables which fitted the bivariable analysis at ≤0.25 level of significance were included in the final model [22]. The strength of association was interpreted using odds ratios with a 95% confidence interval. The Omnibus test and Hosmer-Lemeshow goodness-of-fit were applied to test for model fitness. The level of significance was set at a p-value of <0.05.

### Ethics approval and consent to participate

Ethical clearance was obtained from Ethiopian Public Health Association (EPHA) and the institutional review board (IRB) reviewed and approved the consent procedure (Ref. No: IRB/067/16). An official permission letter was provided from the Ethiopian public health Association (EPHA) to Debre Berhan Comprehensive Specialized Hospital. The procedure and purpose of the study were explained and a discussion was held with the hospital manager and chief clinical officer. In the end, we obtained written informed consent from Debre Berhan comprehensive specialized hospital. Since we used secondary data, informed consent from the participants is not applicable. To maintain confidentiality, identification was not recorded and seriously respected through using registration unit number identifiers. The study was conducted per the declaration of Helsinki Ethical Principles.

## Results

### Socio-demographic and obstetric characteristics

A total of 3322 maternal delivery logbooks and antenatal care files were reviewed. The mean (±SD) age of the participants was 26±5.52 and most women (87%) were in the age group of 18–34 years. About 245 (7.4%) of mothers were grand multipara and 77.3% of them delivered normal birth weight babies. Besides, 5.6% of women delivered at home (See Table 1).

**Table 1:** Socio-demographic and obstetric characteristics of participants.

Variables	Category	Frequency	Percentage (%)
Age	<18	40	1.2
	18–34	2894	87.1
	≥35	388	11.7
Gravidity	Primigravida (1)	1730	52.1
	Multipara (2–4)	1347	40.5
	Grand multipara (≥5)	245	7.4
Parity	Nulliparous	1818	54.7
	1–3	1298	39.1
	≥4	206	6.2
Mode of delivery	Spontaneous vaginal	2114	63.6
	Vacuum	738	22.2
	Forceps	229	6.9
	Cesarean section	211	6.4
	Destructive delivery	30	0.9
Place of delivery	Home	186	5.6
	Institutional	3136	94.4
Number of fetuses	Singleton	3259	98.1
	Twin	63	1.9
Birth weight	<2.5 kg	389	13.7
	2.5–3.99 kg	2272	80.0
	≥4 kg	178	6.3

### Obstetric outcomes

**Fig 1:**
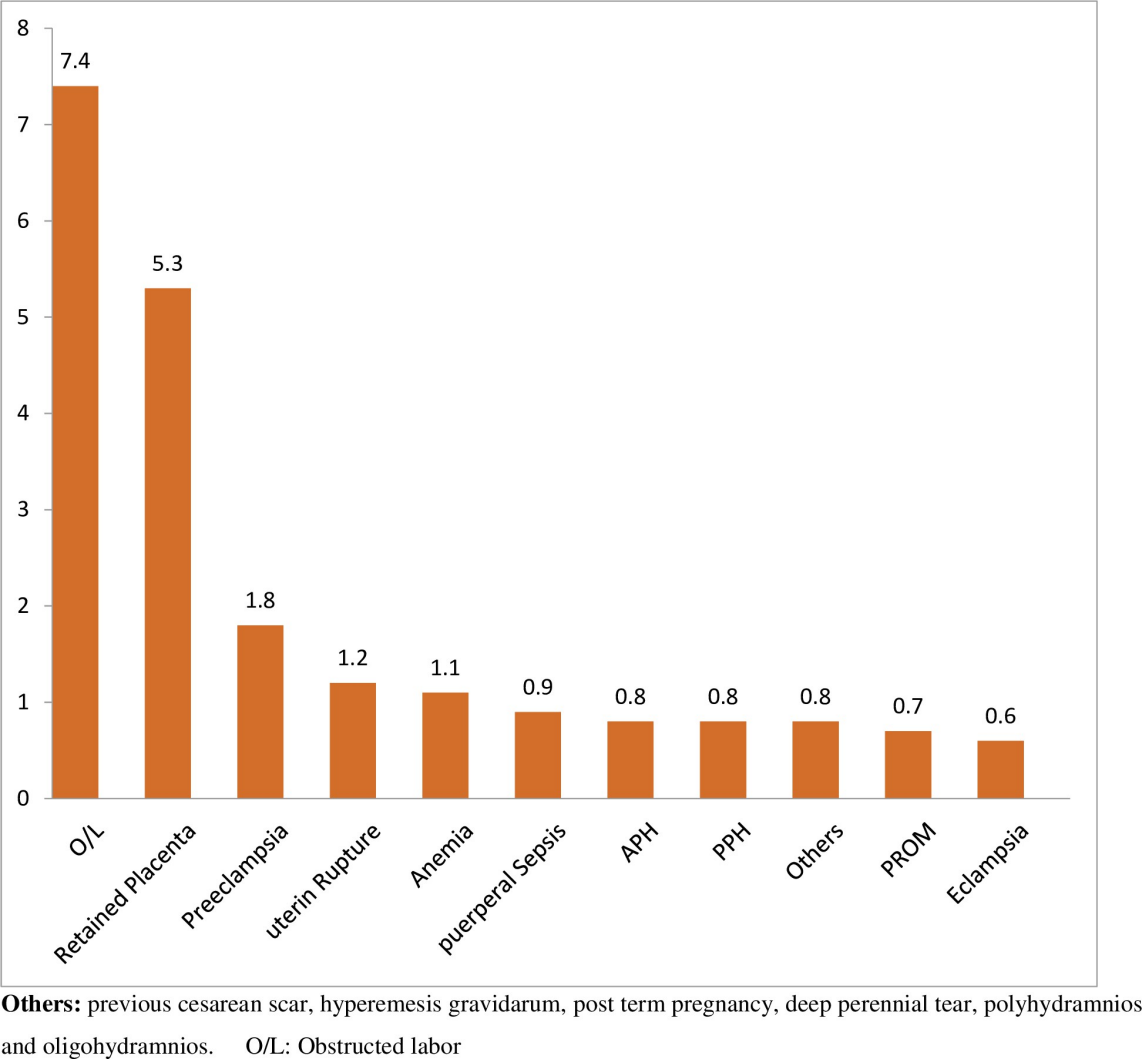
Obstetric complications at Debre Berhan Comprehensive Specialized Hospital, Northeast Ethiopia.

In this study, 941 (28.3%), 95% CI (25.7–30.9) of mothers developed at least one obstetric/fetal adverse outcome. About 322 (9%) women had more than one obstetric complication. Obstructed labor (7.4%), retained placenta (5.3%), and hypertensive disorders of pregnancy (2.4%) were the main obstetric complication (See Fig 1).

There were 29 maternal deaths and the possible causes of death were obstructed labor (51.7%), hemorrhage (44.7%), eclampsia (24.1%), and sepsis (6.9%). The estimated institutional MMR was 873 per 100,000 live births.

### Fetal or neonatal outcomes

**Fig 2:**
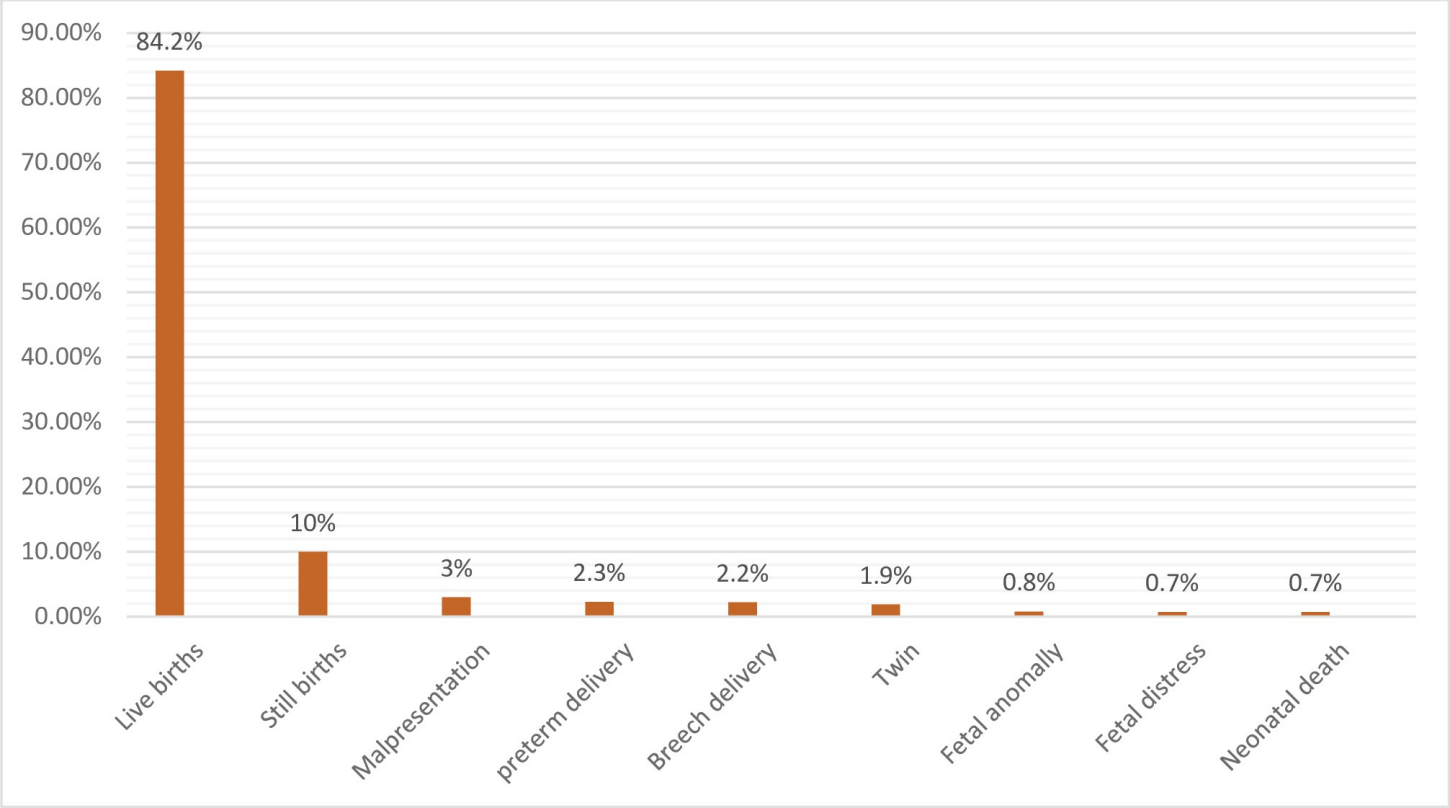
Fetal or neonatal outcome at Debre Berhan Comprehensive Specialized Hospital, Northeast Ethiopia.

About 2795 (84.1%) of newborns were live births. The most frequently identified fetal/neonatal outcomes were stillbirths 332(10%), malpresentation 98(3%), and prematurity 76(2.3%) (See Fig 2). There were 23 early neonatal deaths and the presumed causes of death were prematurity (59.3%), fetal distress (33%), and congenital malformation (12.7%).

### HIV/AIDS assessment

About three-forth (75%) of women were tested for HIV/AIDS status. A total of 150 (4.5%) pregnant women were tested positive, 74 (2.2%) were detected during ANC follow-up and outpatient department (OPD) setting for medical care. The remaining 76 (2.3%) were detected during labor and delivery. Overall, HIV-positive delivery accounted for 125 (3.8%). About 21 (16.8%) of them developed adverse pregnancy outcomes. More than one-fifth 28 (22.4%) of women were on antiretroviral therapy (ART) while 89 (71.2%) women took the antiretroviral drug (ARV prophylaxis) during pregnancy. Overall, 93.6% of HIV-positive mothers were taking ARV drugs for the prevention of mother-to-child transmission (PMTCT). Further, 114 (91.2%) of neonates born from these mothers were provided ARV prophylaxis.

### Factors associated with adverse pregnancy outcome

Bivariable logistic regression analysis was run and fitted into the multivariable logistic regression analysis model. In the multivariable logistic regression analysis, after controlling all other variables, home delivery and low birth weight have shown a statistically significant association with adverse pregnancy outcomes (See Table 2).

**Table 2:** Independent predictors of adverse pregnancy outcome in mothers attending obstetric care at Debre Berhan Comprehensive Specialized Hospital, Northeast Ethiopia.

Variables	Adverse Pregnancy Outcome		COR 95% CI	AOR 95% CI
	Yes (941)	No (2381)		
**Age**				
<18 years	11	29	1.02 (0.51,2.05)	0.92 (0.40,2.12)
18–34 years	776	2118	1	1
≥35 years	154	234	1.72 (1.36,2.17)	1.12 (0.81,1.56)
**Gravidity**				
Primigravida	429	1301	1	1
Multigravida (2–4)	404	943	0.77 (0.66,0.91)	0.66 (0.42,1.06)
Grand multipara (≥5)	108	137	1.85 (1.40,2.44)	1.30 (0.61,2.77)
**Parity**				
Nulliparous	457	1361	1	1
1–3	388	910	1.27 (1.08,1.50)	1.38 (0.61,3.12)
≥4	96	110	2.55 (1.90,3.42)	1.38 (0.84,2.13)
**Place of delivery**				
Home	158	28	8.30 (3.17,14.70)	4.12 (2.30,7.15)*
Institution	760	2376	1	1
**HIV status**				
Positive	21	104	1.50 (0.31,4.80)	1.36 (0.71,3.58)
Negative	920	2277	1	1
**Number of fetuses**				
Singleton	916	2343	1	1
Twin	25	38	1.68 (1.01,2.80)	1.61 (0.92,2.81)
**Fetal weight**				
<2.5 kg	109	280	1.65 (1.38.1.97)	1.63 (1.36,1.96)*
2.5–3.9 kg	435	1837	1	1
≥4 kg	56	122	1.94 (0.94,4.00)	1.50 (0.70,3.23)

* Statistically significant at p-value <0.05.

Mothers who gave birth at home were four times more likely to have adverse pregnancy outcomes compared to those mothers who were attended institutionally (AOR (CI) = 4.12(2.30–7.15). Mothers who gave birth to low-birth-weight neonates (<2.5kg) had a 63% higher risk of developing adverse pregnancy outcomes compared to those who gave birth to 2.5–3.99 kg neonates (AOR (CI) = 1.63 (1.36–1.96) (Table 2).

## Discussion

This study aimed to assess the adverse pregnancy outcomes and its associated factors. The magnitude of adverse pregnancy outcomes was 28.3%, 95% CI (25.7–30.9). The most frequently recorded obstetric complications were obstructed labor (7.4%), retained placenta (5.3%), and hypertensive disorders of pregnancy (2.4%). Whereas stillbirths (10%), malpresentation (3%), and prematurity (2.3%) frequently occurred fetal/neonatal complications. Home delivery and low birth weight were independent predictors of adverse pregnancy outcomes.

In the current study, nine hundred and forty-one (28.3%) mothers developed at least one pregnancy complication. This was comparable with findings in Nepal [7], Bangladesh (25%) [23], and Sub-Saharan Africa [8], in which 27.8%, 25%, and 29.7% of women developed adverse pregnancy outcomes, respectively. A large survey in North Gondar also reported that 28.5% of women developed at least one obstetric complication [15].

However, it was higher than 15.6% of adverse pregnancy outcomes in Zimbabwe [10]. This could be related to the difference in the measurement of outcome variables. The [10] study considered only stillbirth and early neonatal death as adverse pregnancy outcomes. But, the current study, included more adverse events, i.e., obstructed labor, retained placenta, hypertension in pregnancy, low birth weight, stillbirth, macrosomia, malpresentation, and prematurity. In addition, the finding was higher than 24.5% in Hosanna town [11], 21% in Bale Zone [12], and 18.3% in Hawassa town [13]. The variation might be due to the differences in sample size and study setting. The current study was done at the zonal level and hence the magnitude may increase because of an increasing number of referral cases from health centers and primary hospitals. It may be also attributable to the methodological and socio-economic variations, quality of maternal health care services, and facilities in respective study areas.

On the other hand, this finding was lower than the previous studies done in Dessie Referral Hospital [14] and North Wollo Zone [24] where 32.5% and 31.8% of mothers experienced adverse pregnancy outcomes, respectively. The possible explanation could be related to a methodological difference that the previous studies were prospective but in the current study the methodology used was a review of birth records and antenatal care files, and hence this may limit the type and number of pregnancy complications.

In this study, the most frequently recorded obstetric complications were obstructed labor (7.4%), retained placenta (5.3%), anemia (1.1%), and hypertensive disorders of pregnancy (2.4%). Similarly, a systematic chart review in Malawi, South Africa, Uganda, and Zimbabwe found a 4.4% of hypertension in pregnancy [25]. In Southern Ethiopia, hypertensive disorders of pregnancy, APH, PROM, and obstructed labor were the commonest adverse obstetric outcomes [16]. Similarly, in Adigrat Hospital, the main obstetric complications were preterm labor (7.2%), obstructed labor (3.5%), PROM (6%), pre-eclampsia (5.2%), malpresentation (8.7%), APH (2.7%), and PPH (6.7%) [17]. Besides, in Bangladesh, 12% of women reported hemorrhage, 8% sepsis, 11% obstructed labor, and 1% eclampsia [23].

Moreover, stillbirths (10%), malpresentation (3%), and prematurity (2.3%) frequently occurred in fetal/neonatal complications. Similarly, a cohort study in Nepal reported 11% of stillbirths [7]. The most frequently identified adverse birth outcomes in Dessie Referral Hospital were also stillbirth (8.2%), low birth weight (16.7%), preterm (15.2%), and birth defects (8.4%) [14]. Similarly, in Southern Ethiopia, 8.6% of birth outcomes were preterm birth, 9.8% were low birth weight and 8.6% were stillbirths [11]. Further, in Bale Zone, low birth weight (12.2%), preterm birth (8.5%), stillbirth (7.8%), and congenital anomaly (3.1%) were common adverse birth outcomes [12].

This study confirmed a significant association between place of delivery and pregnancy outcomes. Home delivery increased the risk of adverse pregnancy outcomes by fourfold. A population-based study in low-and-middle-income countries (LMICs) found that women with no skilled attendant at birth were at higher risk of having a stillbirth [26]. Another prospective study in Pakistan showed that mothers delivered at home were at risk of postpartum hemorrhage and retained placental tissue [27]. A systematic review and meta-analysis of LMICs concluded that home delivery was significantly associated with neonatal mortality [28]. Besides, in Southern Ethiopia, home delivery increases the risk of adverse perinatal outcomes by 87% [29]. This could be because clean and safe delivery services including cesarean sections and hysterectomies are given at health facilities which in turn prevents trauma, infection, and maternal and neonatal morbidity and mortality. In addition, this finding emphasizes the need of utilizing institutional delivery services. Community mobilization, allying with traditional birth attendants, and addressing socio-cultural factors that affect use of health facilities are vital to increase utilization of institutional delivery services.

Low birth weight was found to be an associated risk factor for adverse pregnancy outcomes. Mothers who gave birth to low-birth-weight neonates had a 63% more risk of developing adverse pregnancy outcomes compared to those who gave birth to normal birth weight neonates. A case-control study in Addis Ababa stated that low birth weight neonates are at increased risk of perinatal mortality [30]. Low birth weight indicates some kind of complication that adversely affects the growth of the fetus. For example, hemorrhage and hypertension disorders of pregnancy may cause abruption placenta, which might result in reduced nutrient and oxygen supply to the growing fetus and may end up in low birth weight or stillbirth [31]. This might be also due to poor maternal nutrition during pregnancy.

### Limitation of the study

Since secondary data were used, some variables of interest such as income, residence, height, alcohol use, and smoking status could not be obtained.

## Conclusion and recommendation

The magnitude of adverse pregnancy outcomes was high. The most frequently recorded obstetric complications were obstructed labor, retained placenta, and hypertensive disorders of pregnancy. Whereas stillbirths, malpresentation, and prematurity frequently occurred fetal/neonatal complications. Place of delivery and birth weight were significant associates of adverse pregnancy outcomes. Institutional delivery, early detection and management of complications, and adequate nutrition and weight gain during pregnancy should be encouraged to minimize the risk of adverse pregnancy outcomes.

## Supporting information

S1 Dataset (SAV)

S1 File English version questionnaire. (DOCX)

## Abbreviations

ANC: Antenatal Care
APH: Antepartum Hemorrhage
EDHS: Ethiopian Demographic Health Survey
HIV: Human Immunodeficiency Virus
MMR: Maternal Mortality Ratio
PPH: Postpartum Hemorrhage
PROM: Premature Rupture of Membrane
WHO: World Health Organization
