# Supplementary material for: Assessment of the adverse pregnancy outcomes and its associated factors among deliveries at Debre Berhan Comprehensive Specialized Hospital, Northeast Ethiopia
Source: PLoS One. 2022 Jul 8;17(7):e0271287. doi: 10.1371/journal.pone.0271287 (PMC9269379; doi:10.1371/journal.pone.0271287)
Supplement: S1 File — (DOCX) [file pone.0271287.s002.docx]

**English version questionnaire**

**Part I: Sociodemographic Information**

| **S. No** | **Questions** | **Responses and coding** |
| --- | --- | --- |
| 101 | Age of the mother | **________**Years |
| 102 | How many times did you become pregnant in your lifetime (Gravidity? | _________ |
| 103 | How many times did you give birth (after 28 completed weeks), Parity? | _________ |
| 104 | Mode of delivery | 1. Spontaneous vaginal 2. Vacuum 3. Forceps 4. Cesarean section 5. Destructive delivery |
| 105 | Place of delivery | 1. Home 2. Institutional |
| 106 | Number of fetuses | 1. Singleton 2. Twin |
| 107 | Birth weight (in KG) | __________________ |

**Part II: Obstetric outcomes**

| **S. No** | **Questions** | **Responses and coding** |
| --- | --- | --- |
| 201 | Obstetric complications | 1. Yes 2. No |
| 201 | Antepartum/Intrapartum/postpartum obstetric outcomes.  Encircle all that apply. | 1. None (Stable) 2. HTN (PE, Gestational, Chronic) 3. APH (Previa, Abruption) 4. PPH 5. PROM 6. Post-term pregnancy 7. Oligohydramnios 8. Polyhydramnios 9. Retained placenta 10. Induction/ Augmentation 11. Anemia 12. CPD/obstructed labor 13. Cord prolapse 14. Uterine rupture 15. Sepsis 16. Maternal death 17. Others_____________ |
| 202 | If the mother died, what was the cause? | _____________________ |

**Part III: Fetal outcomes**

| **S. No** | **Questions** | **Responses and coding** |
| --- | --- | --- |
| 301 | Fetal/neonatal complications | 1. Yes 2. No |
| 301 | The outcome of the newborn | 1. Alive 2. Stillbirth/IUFD 3. Malpresentation 4. Prematurity 5. Breech 6. Twin 7. Congenital anomaly (_________________) 8. Fetal distress 9. Neonatal death |
| 302 | If neonatal death, what was the cause? | ____________________ |

**Part IV: HIV/AIDS assessment**

| **S. No** | **Questions** | **Responses and coding** | **Skip** |
| --- | --- | --- | --- |
| 401 | HIV/AIDS tested | 1. Yes 2. No |  |
| 402 | HIV/AIDS status | 1. Reactive 2. Non-reactive (NR) | If NR, skip the rest. |
| 403 | If positive, when was first detected? | 1. During ANC follow-up 2. Outpatient department 3. At labor and delivery 4. Postpartum period |  |
| 404 | If positive, is she on ART? | 1. Yes 2. No |  |
| 405 | Is she taking ARV prophylaxis during pregnancy? | 1. Yes 2. No |  |
| 406 | Did neonates born from these mothers were provided ARV prophylaxis? | 1. Yes 2. No |  |

**Thank you for your co-operation!!!**
